# Leukocyte telomere length-related genetic variants in *ACYP2* contribute to the risk of esophageal carcinoma in Chinese Han population

**DOI:** 10.18632/oncotarget.16071

**Published:** 2017-03-10

**Authors:** Quan Fang, Lihong Hui, Zhaorui Min, Lifeng Liu, Yuan Shao

**Affiliations:** ^1^ Department of Otorhinolaryngology, The First Affiliated Hospital of Xi’an Jiaotong University, Xi’an, Shaanxi 710061, P.R. China

**Keywords:** esophageal carcinoma, gene polymorphisms, case-control study, *ACYP2*

## Abstract

**Background:**

Short leukocyte telomere length has been associated with significantly increased risk of esophageal carcinoma. A previous genome-wide association study demonstrated that *ACYP2* was associated with leukocyte telomere length. However, the role of *ACYP2* genetic variants on esophageal carcinoma susceptibility is still unknown. Therefore, we investigated whether *ACYP2* polymorphisms have impact on the risk of esophageal carcinoma in Chinese.

**Materials and Methods:**

We conducted a case-control study among 386 cases and 495 healthy controls from northwest China. 14 SNPs in *ACYP2* were selected and genotyped using Sequenom MassARRAY technology. Odds ratios (OR) and 95% confidence intervals (CIs) were calculated by unconditional logistic regression adjusting for age and gender.

**RESULTS:**

We found that 1.34-fold increased risk of esophageal carcinoma is associated with the rs11125529 A allele compared with the rs11125529 C allele (OR=1.29, 95%CI: 1.02-1.62, *p*=0.030) under the additive model, after adjusted by age and gender. We also found rs11896604 and rs17045754 loci increased the esophageal carcinoma risk under the additive model (rs11896604: OR=1.34, 95%CI: 1.03-1.76, *p*=0.032; rs17045754: OR=1.36, 95%CI: 1.03-1.80, *p*=0.028). One main linkage block was observed across the locus. This block was comprised of seven closely linked SNPs: rs1682111, rs843752, rs10439478, rs843645, rs11125529, rs843711 and rs11896604. The haplotype analysis detected that haplotype “TTCTATG” increased the risk of esophageal carcinoma (OR=1.38, 95%CI: 1.04-1.82, *p*=0.025).

**Conclusion:**

In conclusion, *ACYP2* gene may be associated with an increased risk of esophageal carcinoma in Chinese Han populations. Future studies to address the biological function of this polymorphism in the development of esophageal carcinoma are warranted.

## INTRODUCTION

Esophageal carcinoma (EC) is one of the most common cancers in our country, and the survival rate of five year is less than 15%, its clinical effect is still limited at present [1]. In China, the incidence of esophageal carcinoma is 21.88/105, and higher than western countries [2]. Esophageal carcinoma according to histological type can be divided into esophageal squamous cell carcinoma, adenocarcinoma and undifferentiated carcinoma. Among them, esophageal squamous cell carcinoma accounts for 90%. In terms of lifestyle, smoking has been identified can cause esophageal cancer, especially smoking and excessive drinking can significantly increase the risk of esophageal carcinoma [3, 4]. Genetic factors also have an impact on the occurrence of esophageal carcinoma.

Cui et al. [5] found two significant susceptibility genes, ADH1B and ALDH2 in 1070 cases of Japanese esophageal carcinoma and 2836 controls. Wang et al. [6] found that *PLCE1* gene and *C20orf54* gene associated with ESCC. GWAS study of Wu et al. in Chinese population of esophageal cancer, and seven susceptibility sites for esophageal cancer were found in five regions (5q11 rs10052657, 21q22 rs2014300, 6p21 rs10484761, 10q23 rs2274223 and 12q24 rsl1066015, rs2074356, rs11066280) [7]. Abnet et al. [8] found that rs2274223 in PLCE1 associated with ESCC. Beyond that, genes associated with susceptibility to ESCC mainly contain metabolizing enzyme-associated genes (*CYP450*, *GSTP1*, *ALDH2*), nucleotide excision repair genes (*XRCC1*, *XPD*, *h*OGG1), tumor suppressor genes, oncogenes (*p53*, *ECRG1*, *ECRG2*, *cyclin*D1), cytokines (*IL6*, *IL18*, *IL12*), matrix metalloproteinase (MMPs).

Telomere contains repeating nucleotide sequences and a related terminal protein complex that plays a key role in maintaining chromosome integrity and stability, as well as telomere shortening involved in the carcinogenesis and progression of malignant tumors [9]. Associations with telomere length have been reported for cancers of the digestive system [10, 11]. Du et al. [12] found that telomere length effected the risk of esophageal squamous cell carcinoma risk, telomere too short or too long to increase the risk of esophageal cancer. A genome-wide meta-analysis identified seven loci, including *ACYP2* rs11125529, associated with mean leukocyte telomere length [13]. However, the role of *ACYP2* gene on esophageal carcinoma susceptibility is still unknown. So, we want to examined whether the *ACYP2* gene polymorphism have impact on the risk of esophageal carcinoma in Chinese population.

## RESULTS

The characteristics of these populations are summarized in Table 1. We included 386 esophageal carcinoma patients, which consisted of 308 males and 78 females with a mean age of 60.68±8.95 years at the time of diagnosis. The 495 healthy controls were recruited, included 180 males and 315 females with a mean age of 54.48±9.44 years. It exist differences between gender and age distribution between the case and control groups.

**Table 1:** Characteristics of esophageal carcinoma cases and controls

	Case	Control	*p*
Gender	386	495	*p* <0.001
female	78	315	
male	308	180	
Mean Age, year	60.68±8.95	54.48±9.44	*p* <0.001

*p* < 0.05 indicates statistical significance.

14 SNPs in the *ACYP2* gene were analyzed in our study. Chromosomal, position, HWE *p* value, minor allele frequency for all the SNPs are shown in Table 2. The rs843740 and rs12615793 was cut off at 5% HWE *p* level.

**Table 2:** Frequency distributions of alleles and their associations with esophageal carcinoma

SNP rs#	Chromosome	Position	Alleles A/B	Gene(s)	MAF-Case	MAF-Control	HWE *p*-value	OR (95%CI)	*p*-value
rs6713088	2	54345469	G/C	ACYP2	0.41	0.40	0.778	1.08(0.89-1.31)	0.448
rs12621038	2	54391113	T/C	ACYP2	0.47	0.46	0.786	1.05(0.87-1.27)	0.598
rs1682111	2	54427979	A/T	ACYP2	0.32	0.30	0.832	1.08(0.88-1.32)	0.482
rs843752	2	54446587	G/T	ACYP2	0.24	0.27	0.569	0.87(0.7-1.08)	0.219
rs10439478	2	54459450	C/A	ACYP2	0.44	0.43	0.927	1.02(0.84-1.23)	0.860
rs843645	2	54474664	G/T	ACYP2	0.23	0.26	1.000	0.86(0.69-1.07)	0.166
rs11125529	2	54475866	A/C	ACYP2	0.21	0.18	0.092	1.23(0.97-1.56)	0.093
rs12615793	2	54475914	A/G	ACYP2	0.23	0.19	0.042	1.29(1.02-1.62)	0.030
rs843711	2	54479117	T/C	ACYP2	0.46	0.45	0.124	1.04(0.86-1.26)	0.689
rs11896604	2	54479199	G/C	ACYP2	0.23	0.19	0.249	1.21(0.96-1.52)	0.112
rs843706	2	54480369	A/C	ACYP2	0.47	0.46	0.069	1.03(0.85-1.25)	0.745
rs17045754	2	54496757	C/G	ACYP2	0.21	0.18	0.297	1.18(0.93-1.5)	0.166
rs843740	2	54499344	A/G	ACYP2	0.40	0.46	0.000	0.78(0.64-0.94)	0.010
rs843720	2	54510660	G/T	ACYP2	0.37	0.34	0.422	1.16(0.95-1.41)	0.146

HWE, Hardy-Weinberg Equilibrium; MAF, minor allele frequency; ORs, odds ratios; CI, confidence interval;

*p* < 0.05 indicates statistical significance.

Further genetic models analyses used logistic test, adjusted by age and gender, we found that rs11125529, rs11896604 and rs17045754 loci increased the esophageal carcinoma risk under the additive model (rs11125529: OR=1.34, 95%CI: 1.01-1.77, *p*=0.04; rs11896604: OR=1.34, 95%CI: 1.03-1.76, *p*=0.032; rs17045754: OR=1.36, 95%CI: 1.03-1.8, *p*=0.028) (Table 3).

**Table 3:** The association between *ACYP2* gene polymorphisms and esophageal carcinoma adjusted by age and gender

SNP	model	genotype	control	case	OR(95%CI)	*p*	OR(95%CI)	*p*
rs11125529	genotype model	CC	327	234	1		1	
		AC	158	131	1.16(0.87-1.54)	0.313	1.3(0.94-1.8)	0.114
		AA	10	15	2.10(0.93-4.75)	0.076	2.02(0.82-4.97)	0.127
	dominant model	CC	327	234	1		1	
		AC-AA	168	146	1.21(0.92-1.60)	0.171	1.35(0.98-1.85)	0.063
	recessive model	CC-AC	485	365	1		1	
		AA	10	15	1.99(0.89-4.49)	0.096	1.84(0.75-4.51)	0.18
	additive model	-	-	-	1.24(0.97-1.59)	0.084	1.34(1.01-1.77)	0.04
rs11896604	genotype model	CC	317	226	1		1	
		CG	164	137	1.17(0.88-1.56)	0.274	1.36(0.99-1.88)	0.061
		GG	14	17	1.70(0.82-3.53)	0.152	1.73(0.77-3.89)	0.188
	dominant model	CC	317	226	1		1	
		CG-GG	178	154	1.21(0.92-1.60)	0.168	1.39(1.02-1.91)	0.038
	recessive model	CC-CG	481	363	1		1	
		GG	14	17	1.61(0.78-3.01)	0.196	1.55(0.69-3.45)	0.288
	additive model	-	-	-	1.22(0.96-1.59)	0.104	1.34(1.03-1.76)	0.032
	genotype model	GG	325	233	1		1	
rs17045754		CG	157	127	1.13(0.85-1.50)	0.412	1.33(0.96-1.85)	0.086
		CC	13	16	1.71(0.81-3.64)	0.158	2.02(0.86-4.74)	0.106
	dominant model	GG	325	233	1		1	
		CG-CC	170	143	1.17(0.89-1.55)	0.261	1.39(1.01-1.90)	0.044
	recessive model	GG-CG	482	360	1		1	
		CC	13	16	1.65(0.78-3.47)	0.186	1.83(0.79-4.25)	0.161
	additive model	-	-	-	1.19(0.93-1.51)	0.160	1.36(1.03-1.80)	0.028

ORs, odds ratios; CI, confidence interval; *p* < 0.05 indicates statistical significance.

**Figure 1:**
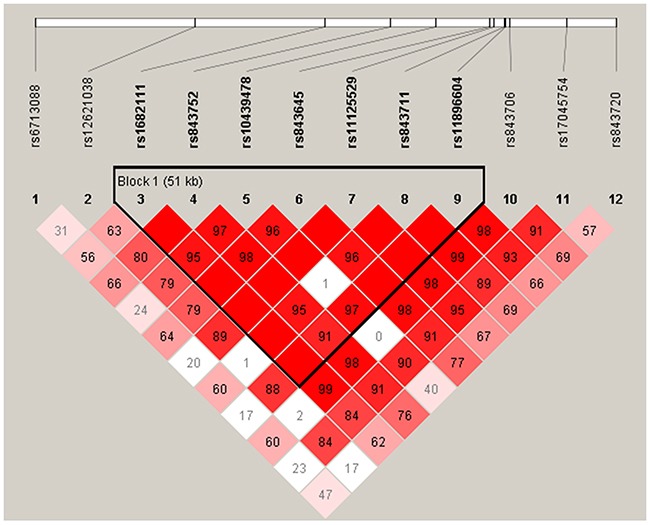
Haplotype block map for all the SNPs of the *ACYP2* gene A standard color scheme is used to display LD with bright red for very strong LD (LOD=2, D’= 1), white for no LD (LOD<2, D’<1), pink red (LOD=2, D’<1), and blue (LOD<2, D’=1) for intermediate LD.

D ’and r^2^ were used to measure the degree of linkage disequilibrium between the two SNPs. D’ confidence intervals were used to classify the haplotypes. One main linkage block was observed across the locus (Figure 1). This block was comprised of seven closely linked SNPs: rs1682111, rs843752, rs10439478, rs843645, rs11125529, rs843711, rs11896604. The haplotype analysis detected that haplotype “TTCTATG” increased the risk of esophageal carcinoma (OR=1.38, 95%CI: 1.04-1.82, *p*=0.025) (Table 4).

**Table 4:** The haplotype frequencies of gene polymorphisms and esophageal cancer risk

Gene	SNPs	Haplotype	Crude analysis		Adjusted analysis	
			OR(95%CI)	*p*	OR(95%CI)	*p*
ACYP2	rs1682111|rs843752|rs10439478|rs843645|rs11125529|rs843711|rs11896604	TGAGCTC	0.82(0.65-1.03)	0.087	0.81(0.63-1.04)	0.096
		ATATCCC	1.08(0.87-1.32)	0.493	0.99(0.79-1.25)	0.939
		TTCTCCC	0.84(0.67-1.06)	0.147	0.88(0.67-1.14)	0.316
		TTCTATG	1.28(1-1.64)	0.050	1.38(1.04-1.82)	0.025
		TTCTCTG	0.58(0.2-1.7)	0.324	1.23(0.36-4.19)	0.746

ORs, odds ratios; CI, confidence interval;

*p* < 0.05 indicates statistical significance.

## DISCUSSION

We adopted case-control method to explore the influence of *ACYP2* genetic polymorphisms on esophageal carcinoma. We found that rs11125529, rs11896604 and rs17045754 loci increased the esophageal carcinoma risk Chinese Han population.

The *ACYP2* gene encodes an 11 kDa acylphosphatase, a small cytoplasmic enzyme that is widely distributed in vertebrate tissue in two different isoenzyme forms. Acyl phosphatase is one of the smallest molecular weight proteins found in the present study, which catalyze the hydrolysis of acyl phosphate compounds *in vitro*. The study of the acyl phosphatase in vertebrates indicates that it is related to many important biological processes, such as glycolysis pathway, apoptosis, Na+, K+ and Ca2+ ion pump control, etc. The main function of ACYP2 is closely related to pyruvate metabolism, cell differentiation and apoptosis [14]. Therefore, the mutation of ACYP2 may be related to tumorigenesis.

There are few studies on ACYP2 gene and disease. One study found that overexpression of ACYP triggers cell differentiation of SH-SY5Y neuroblastoma cells [15]. In colorectal cancer, ACYP is also involved in the metastasis of human colorectal cancer cell line [16]. In our research, we also found that rs11896604 and rs17045754 loci on ACYP2 gene increased the esophageal carcinoma risk. But, it is unclear that this site is how to affect the incidence of esophageal cancer. In the future, we will adopt molecular biology and cell biology method to study the mechanism of action.

Du et al. [12] found that telomere length effected the risk of esophageal squamous cell carcinoma risk, telomere too short or too long to increase the risk of esophageal cancer. Codd et al. by a large case-control study found 7 genes that affect telomere length (TERC, TERT, NAF1, OBFC1, ZNF208, RTEL1 and ACYP2), they determine the ACYP2 rs11125529 locus mutation shortened telomere length in European populations [13]. In our study, we found that *ACYP2* rs11125529 increased the risk of esophageal cancer. Telomere contains repeating nucleotide sequences and a related terminal protein complex that plays a key role in maintaining chromosome integrity and stability, as well as telomere shortening involved in the carcinogenesis and progression of malignant tumors [9]. So, we assume that *ACYP2* gene affecting the incidence of esophageal cancer through impact the telomere length.

In our research, we also found that rs11896604 and rs17045754 loci on *ACYP2* gene increased the esophageal carcinoma risk. The rs11896604 and rs17045754 loci on the ACYP2 gene were found to be associated with ischemic stroke in a case-control study of 300 patients with ischemic stroke and 300 healthy individuals [17]. In a study of ACYP2 and high-altitude pulmonary edema, two sites, rs11896604 and rs12615793, were found to reduce the risk of high-altitude pulmonary edema [18]. But, it is unclear that this site is how to affect the incidence of esophageal cancer. In the future, we will adopt molecular biology and cell biology method to study the mechanism of action.

Because the information of patient is not complete, there is no analysis of the impact of these factors (Cigarette smoking and alcohol drinking) on esophageal cancer. So the association between *ACYP2* gene polymorphisms and drinking and smoking status in esophageal carcinoma need to be evaluated in future studies.

In summary, this study results provided new evidence that *ACYP2* gene was associated with esophageal carcinoma in the Chinese Han population from northwest China, which may serve as a prognostic biomarker for esophageal carcinoma among Chinese population. Future study will focus on the function of *ACYP2* in esophageal carcinoma patients, leading to the better prevention or early detection and better prognosis for esophageal carcinoma.

## MATERIALS AND METHODS

### Study participants

We adopted case-control method to research the association of *ACYP2*, telomere related genes, on esophageal carcinoma risk. From 2014 July to 2015 October, 386 participants with esophageal carcinoma at the first affiliated Hospital of Xi’an Jiaotong University were recruited. All esophageal carcinoma patients were diagnosed according to X-ray barium meal examination. Observe the esophageal peristalsis, the tension of the wall, the changes of the esophageal mucosa, the filling defect of the esophagus and the degree of obstruction. Esophageal peristalsis pause or reverse peristalsis, the esophageal wall local stiffness could not be fully dilated, esophageal mucosa disorder, disruption and destruction, esophageal stenosis, irregular filling defect or ulcer and esophageal fistula, and esophageal axial abnormalities are important symptom for esophageal carcinoma. Patients with any previous cancers or autoimmune diseases, chemotherapy or radiotherapy were excluded from the study. Meanwhile 495 healthy blood donors were selected as control group in the first affiliated Hospital of Xi’an Jiaotong University.

All of the participants signed an informed consent agreement. The Human Research Committee for Approval of Research Involving Human Subjects, the first affiliated Hospital of Xi’an Jiaotong University approved the use of human tissue in this study.

### SNP selection and genotyping

Among the 14 SNPs were selected, rs11125529 was chosen from previously published polymorphisms associated with telomere length [13], others were randomly chosen from the published gene *ACYP2* associated with telomere length, with minor allele frequencies >5% in the HapMap Chinese Han Beijing population. We used the GoldMag-Mini Whole Blood Genomic DNA Purification Kit (GoldMag Co. Ltd. Xi’an City, China) extracted from whole blood. Using a NanoDrop 2000 (Gene Company Limited) were measured DNA concentrations. We used Sequenom MassARRAY Assay Design 3.0 Software to design a Multiplexed SNP MassEXTEND assay [19]. Sequenom MassARRAY RS1000 was used for genotyping, and the related data were managed using Sequenom Typer 4.0 Software [19, 20]. Laboratory personnel were blinded to the genotyping results of all samples.

### Statistical analysis

Data analysis was performed using Microsoft Excel (Redmond, WA, USA) and SPSS 19.0 statistical package (SPSS, Chicago, IL, USA). All *p* values were two-sided, and p <0.05 was indicated statistical significance. Each SNP frequency in the control subjects was assessed for departure from Hardy–Weinberg Equilibrium (HWE) using an exact test. We calculated genotype frequencies of cases and controls using a χ^2^ test [21]. Odds ratios (ORs) and 95 % confidence intervals (CIs) were determined using unconditional logistic regression with adjustment for age and sex [22].

Fourgenetic models (genotype, dominant, recessive, and additive model) were performed using PLINK software (https://www.cog-genomics.org/plink2), to characterize the potential association of each ACYP2 polymorphism with the risk of esophageal carcinoma. Finally, we used Haploview software package (version 4.2) to do haplotype analysis used 495 control samples. Firstly, we make linkage disequilibrium analysis. Using the parameter D’ and r2 to measure the degree of linkage disequilibrium between the two SNPS loci. Using D’ confidence interval method divided haplotype block haplotype block. |D’|≤1, the more close to 1, the higher the level of linkage disequilibrium between sites; R^2^≤1, the more close to 1, the higher the level of linkage disequilibrium between the loci. The Odds ratios (ORs) and 95% confidence intervals (CIs) of haplotype were determined using unconditional logistic regression with adjustment for age and sex [23, 24].
